# Supplementary material for: Assessment of the Biocompatibility Ability and Differentiation Capacity of Mesenchymal Stem Cells on Biopolymer/Gold Nanocomposites
Source: Int J Mol Sci. 2024 Jun 30;25(13):7241. doi: 10.3390/ijms25137241 (PMC11242884; doi:10.3390/ijms25137241)
Supplement: Supplementary file 1 [file ijms-25-07241-s001.zip › ijms-3023674-supplementary.pdf]

Assessment the Biocompatibility Ability and Differentiation Capacity of Mesenchymal Stem Cells on Biopolymer/Gold Nanocomposites

Supplementary data

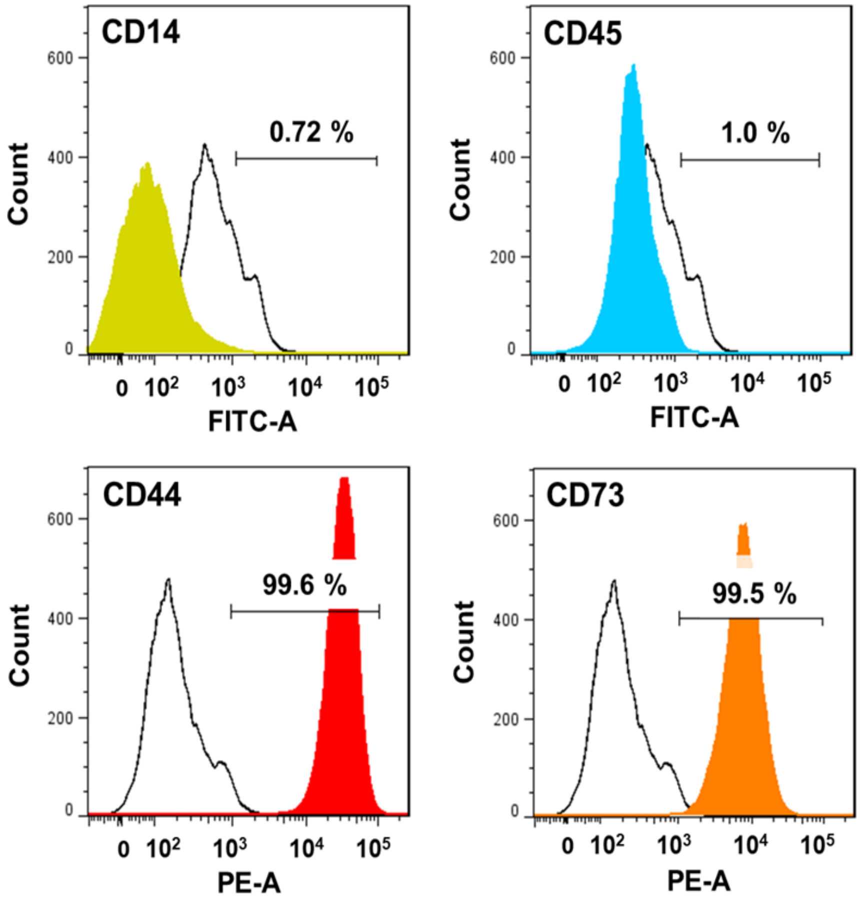

Figure S1. Phenotype characterization of MSCs. The antibodies were conjugated with Fluorescein Isothiocyanate (FITC) and Phycoerythrin (PE), with the following markers of CD14-FITC, CD45-FITC, CD44-PE, and CD73-PE and analyzed by flow cytometry. All the results are representative of one of three independent experiments.

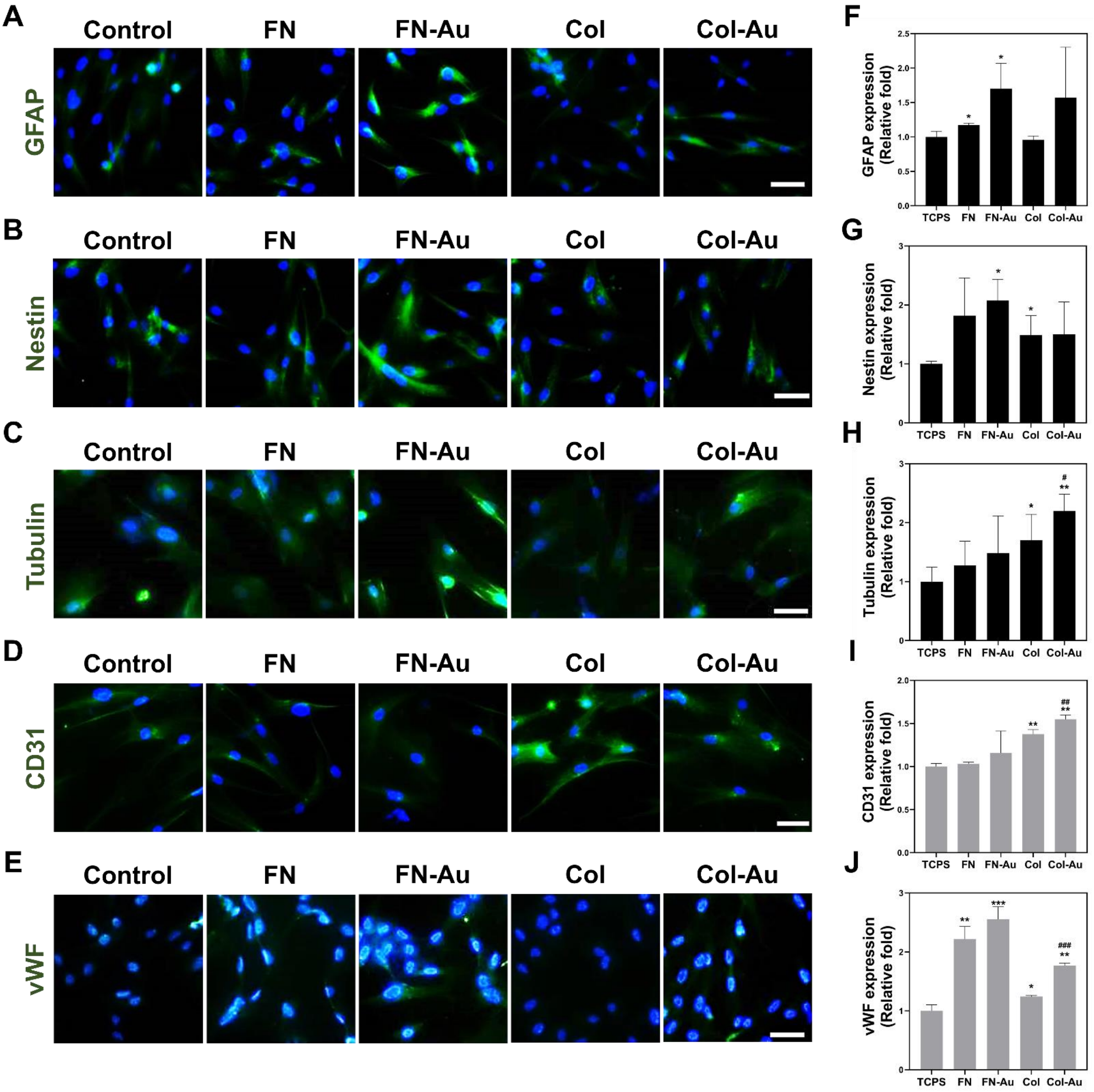

Figure S2. The multiple differentiation capability of MSCs in various materials at day 3. **(A-E)** The MSCs were stained with five primary antibodies and then conjugated with secondary FITC-immunoglobin secondary antibodies. **(F-H)** The expression of GFAP, Nestin, and Tubulin were used to assess neural differentiation. Scale bar= 20 µm. **(I, J)** vWF and CD31 endothelial markers were for endothelial differentiation. *$p < 0.05$, **$p < 0.01$, ***$p < 0.001$: compared to the control. #$p<0.05$, ##$p<0.01$, ###$p<0.001$: compared to the pure substances (FN and Col).

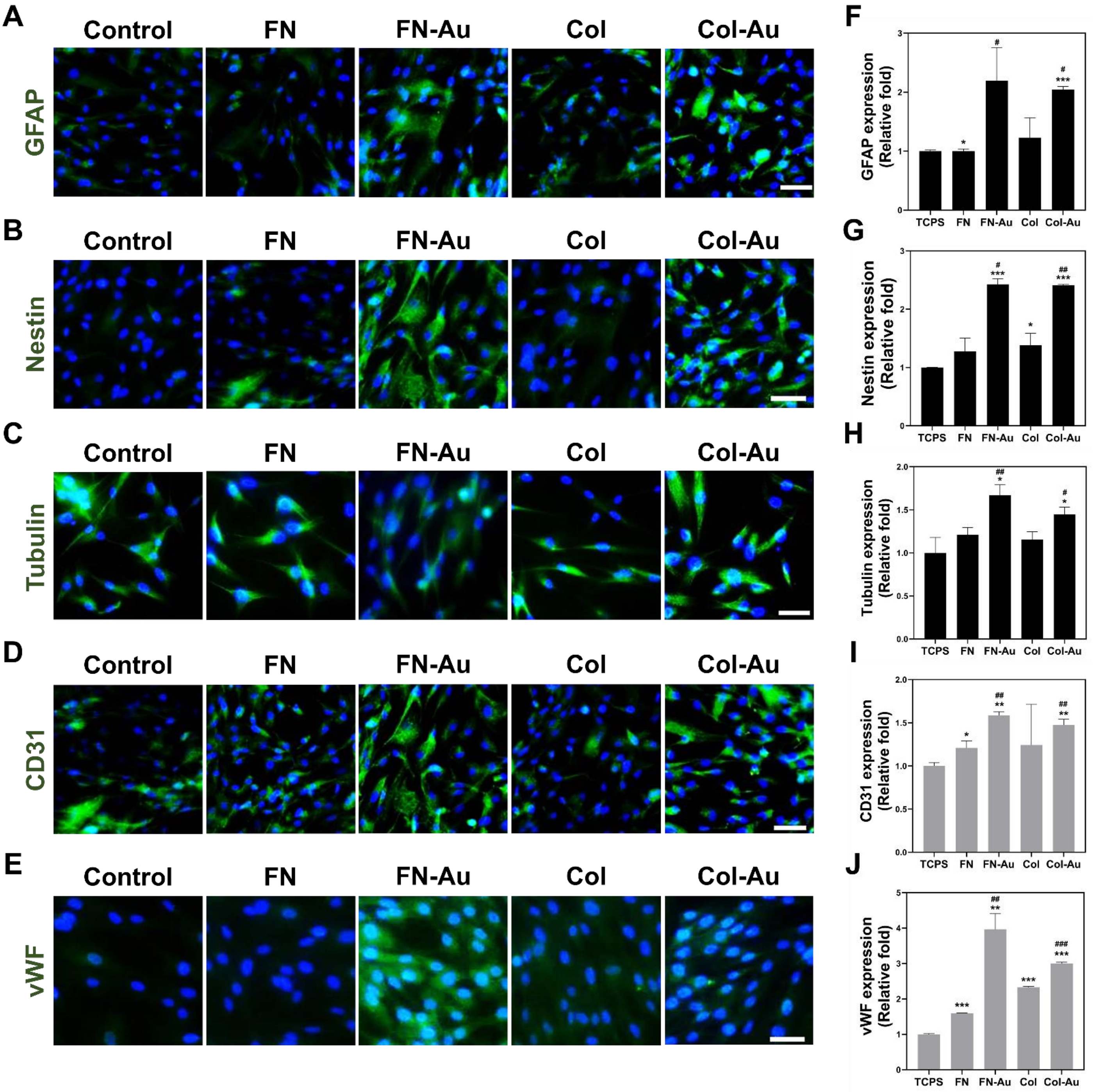

Figure S3. The multiple differentiation capability of MSCs in various materials at day 5. **(A-E)** The MSCs were stained with five primary antibodies and then conjugated with secondary FITC-immunoglobin secondary antibodies. Scale bar= 20 μm. **(F-H)** The expression of GFAP, Nestin, and Tubulin were used to assess neural differentiation. **(I, J)** vWF and CD31 endothelial markers were for endothelial differentiation. *p < 0.05, **p < 0.01, ***p < 0.001: compared to the control. #p<0.05, ##p<0.01, ###p<0.001: compared to the pure substances (FN and Col).

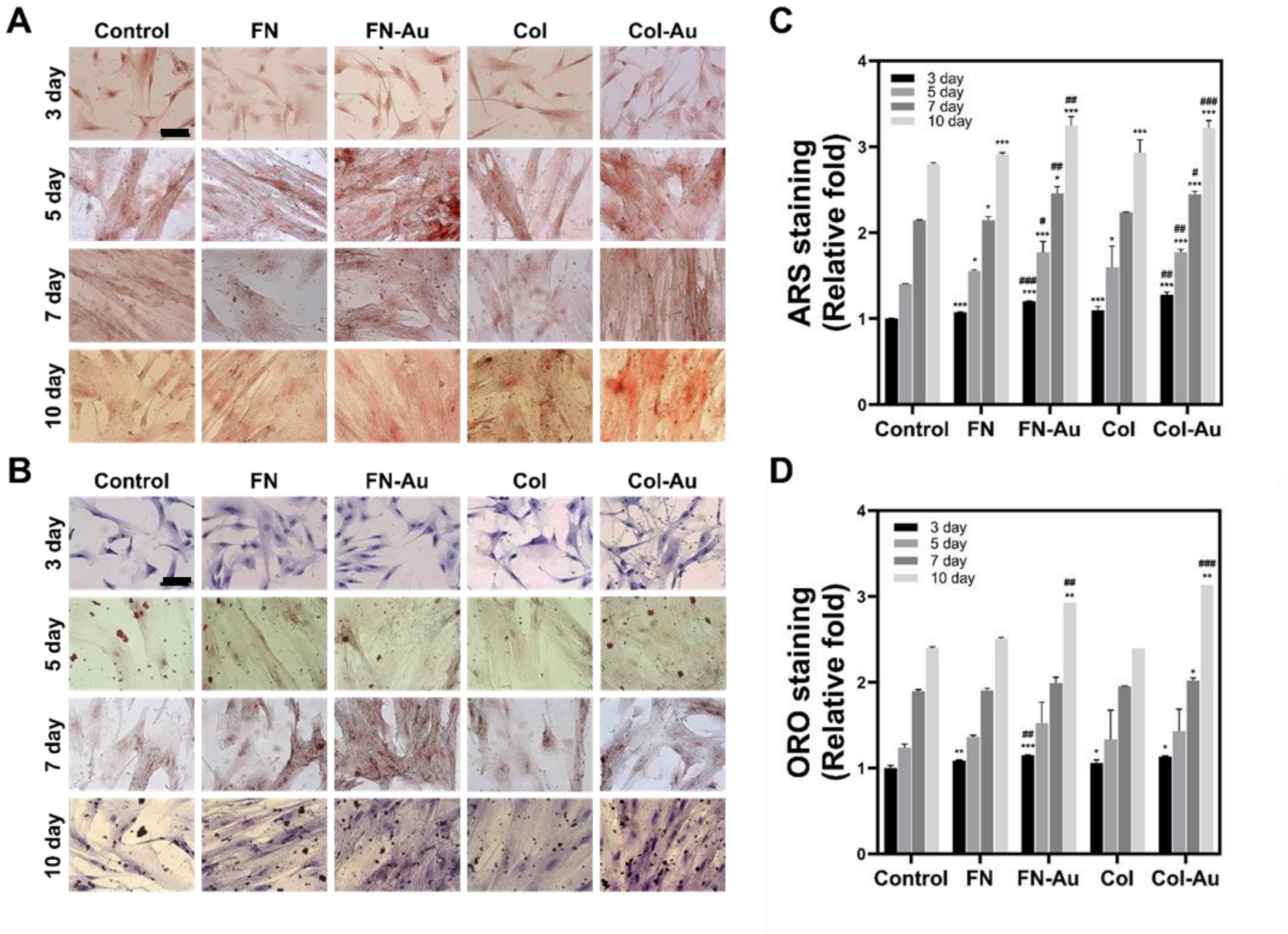

Figure S4. Assessments of osteoblast and adipocyte differentiation using ARS and ORO staining between day 3 to day 10. **(A)** Calcium deposition of MSCs was stained with ARS staining. **(B)** The neutral lipids in the MSCs were stained with ORO staining. Scale bare=50 μm. **(C, D)** The semi-quantitative results for calcium deposition and neutral lipid. *p < 0.05, **p < 0.01, ***p < 0.001: compared to the control. #p<0.05, ##p<0.01, ###p<0.001: compared to the pure substances (FN and Col).
